# Supplementary material for: High-throughput single-cell rheology in complex samples by dynamic real-time deformability cytometry
Source: Nat Commun. 2019 Jan 24;10:415. doi: 10.1038/s41467-019-08370-3 (PMC6346011; doi:10.1038/s41467-019-08370-3)
Supplement: Supplementary file 1 — Supplementary Information [file 41467_2019_8370_MOESM1_ESM.pdf]

Supplementary Information

High-throughput single-cell rheology in complex samples by dynamic real-time deformability cytometry

Fregin et al.

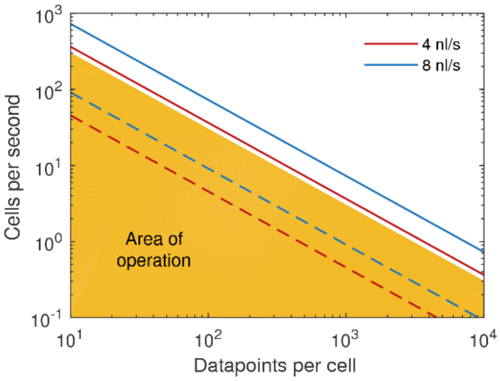

Supplementary Figure 1: Throughput of dynamic real-time deformability cytometry. Solid lines show minimum frame rates for capturing more than 80 % of the cells in 0.5 % MC buffer (0.5 % methylcellulose in PBS-/-) while dashed lines represent calculations for 1.0 % MC buffer. In both estimations equal magnitudes in cell deformation have been assumed.

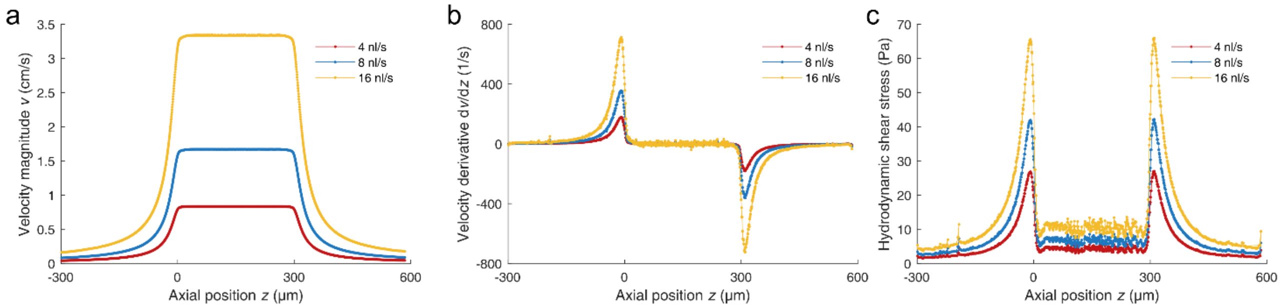

Supplementary Figure 2: Velocity profile and hydrodynamic shear stress along channel center line. a) Velocity magnitude (all vector components) for three typical flow rates of 4 nl s^{-1} (red), 8 nl s^{-1} (blue) and 16 nl s^{-1} (yellow) reaching a plateau inside 30 µm x 30 µm channel. The channel starts at z = 0 µm and ends at z = 300 µm. b) Velocity gradient in direction of flow. c) Hydrodynamic shear stress showing a peak at the channel inlet (z = 0 µm) and channel outlet (z = 300 µm) and a constant plateau inside the constriction. Calculations have been done without a cell using finite element method simulations in COMSOL Multiphysics 5.3a.

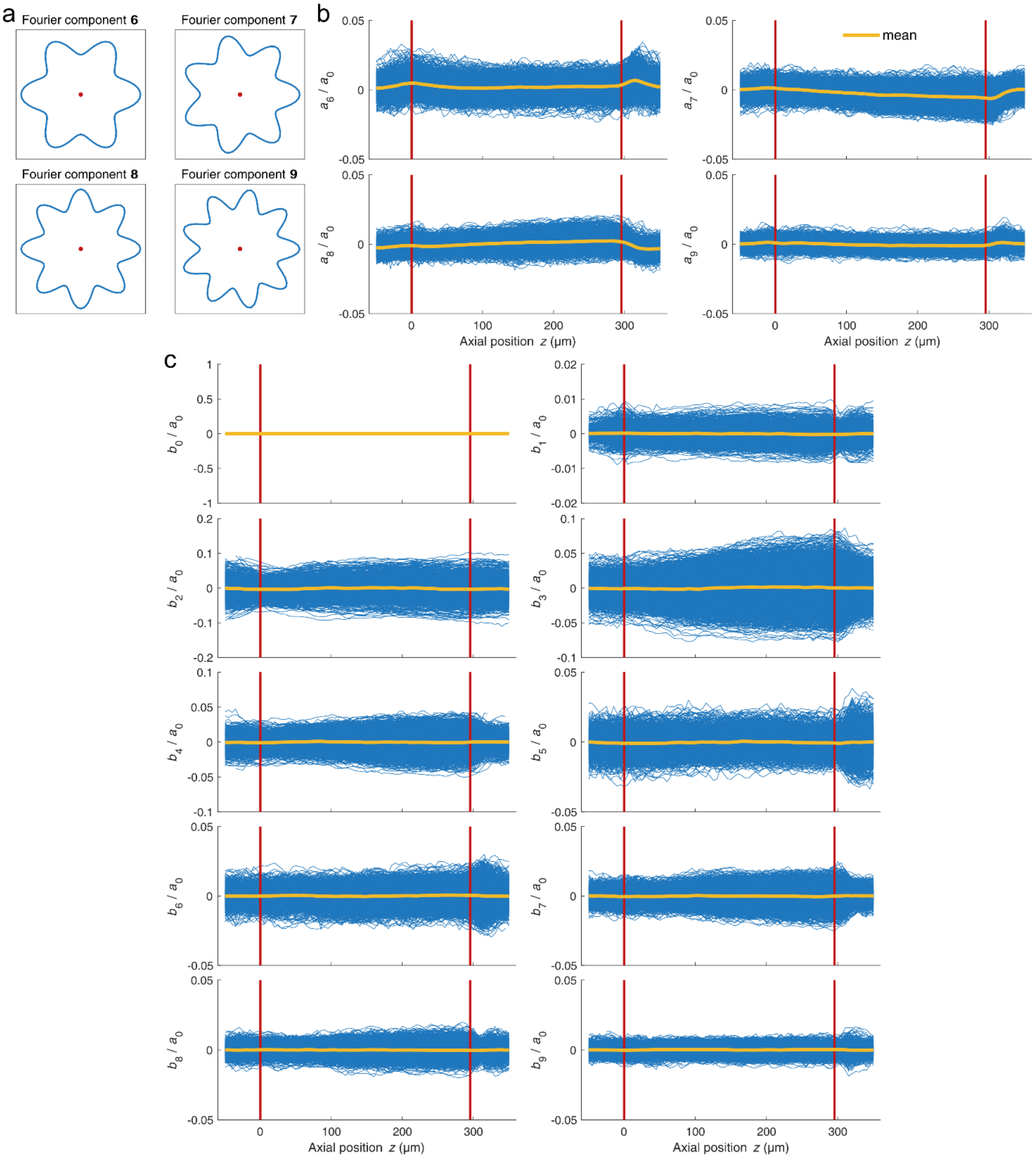

Supplementary Figure 3: Fourier decomposition of cell shape. a) Shape modes for components six to nine including component zero. b) Shape mode analysis (components six to nine) applied to dynamic measurements of HL60 cells arranged for even and odd coefficients. Each blue trace represents the amplitude of a Fourier coefficient of a single cell over the axial position z in the channel. The yellow lines indicate the mean over all traces whereas the red vertical lines visualize the inlet and the outlet position. c) Shape mode analysis as in (b) for first ten Fourier coefficients b_k which represent the angular position of the cell inside the channel. Because the cells are oriented in the direction of flow, the mean over all b_k is close to zero. Measurements have been carried out in a 30 µm x 30 µm channel at a flow rate of 8 nl s^{-1} (n=1,580 cells). The mean shear rate of 5,100 s^{-1} and the mean shear stress of 142 Pa on the cell surface has been derived from finite element method simulations considering the full microfluidic geometry.

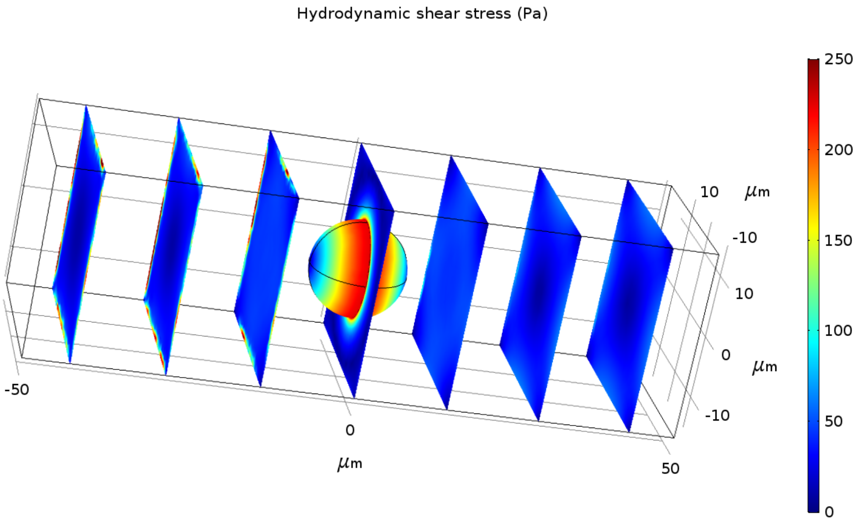

Supplementary Figure 4: Finite element method simulation of steady-state surface stress. The graph shows the shear stress distribution on a sphere of a diameter of 15.9 µm flowing through a channel of 30 µm x 30 µm cross-section. Calculations have been conducted under steady-state conditions for a flow rate of 8 nl s^{-1}, a shear rate of 5,100 s^{-1} and the average shear stress on the sphere surface has been calculated to 142 Pa. Finite element method simulations have been carried out using COMSOL Multiphysics 5.3a.

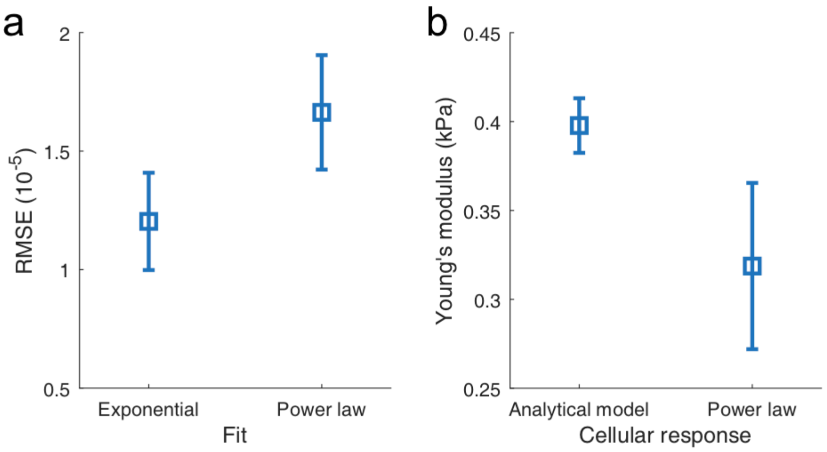

Supplementary Figure 5: Comparison between linear viscoelastic model and power law rheology. a) Root-mean-square-error (RMSE) of exponential (n=3,382 cells) versus power law (n=3,344 cells) fit to reconstructed mean deformation d_{channel} versus time trace for three biological replicates. b) Young's modulus derived from analytical model assuming linear viscoelasticity (analytical model) and power-law rheology (power law). For power-law rheology cellular strain was approximated to 10 % from relative surface displacement and an apparent Young's modulus of 319 ± 47 Pa and a fluidity of β=0.42 ± 0.02 is obtained from a fit to our data. Strains of 8 % and 12 % result in an apparent Young's modulus of 398 ± 58 Pa and 266 ± 39 Pa, respectively while the fluidity remains the same. Measurements have been carried out in a 30 µm x 30 µm channel at flow rate of 8 nl s^{-1}. The mean shear rate of 5,100 s^{-1} and the mean shear stress of 142 Pa on the cell surface has been derived from finite element method simulations considering the full microfluidic geometry. Error bars represent standard error of the mean.

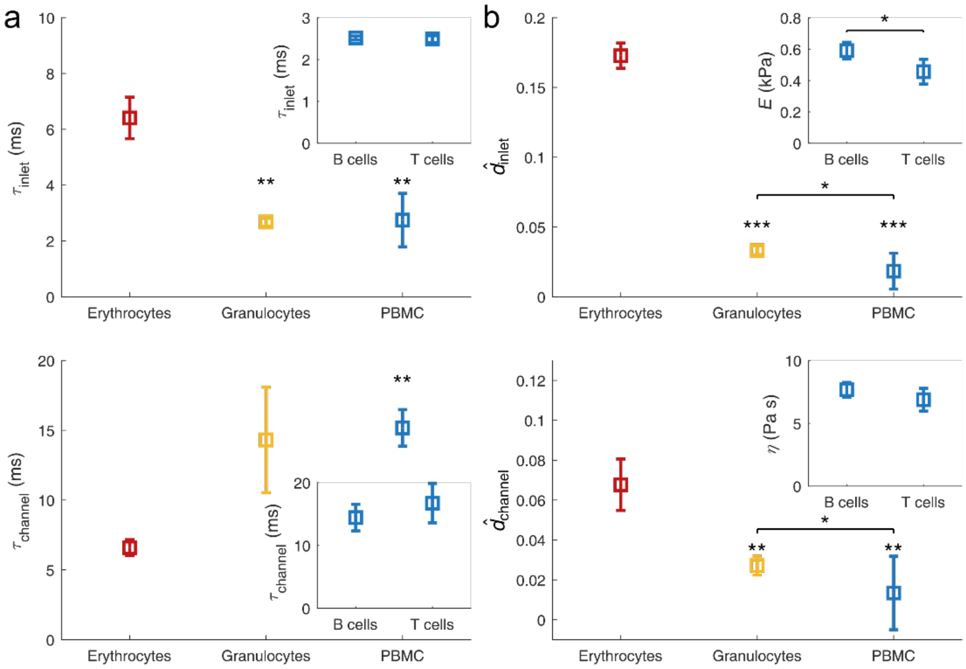

Supplementary Figure 6: Single cell rheology on peripheral blood cells. Statistical analysis comparing *n*=381 erythrocytes (red), *n*=243 granulocytes (yellow), *n*=130 peripheral blood mononuclear cells (PBMC) (blue), *n*=64 B-cells and *n*=348 CD4+ T-cells of three biological replicates for (a) characteristic time scales τ_{inlet} (top) and τ_{channel} (bottom) and (b) peak deformation $\hat{d}_{\text{inlet}}$ (top) and $\hat{d}_{\text{channel}}$ (bottom). The insets show a comparison between B- and T-cells for (a) τ_{inlet} and τ_{channel} and for (b) E and η. Experiments were carried out in a 20 µm x 20 µm channel at a flow rate of 4 nl s^{-1}. The mean shear rate of 9,700 s^{-1} and the mean shear stress of 216 Pa on the cell surface (granulocytes and PBMCs) as well as the mean shear rate of 8,600 s^{-1} and the mean stress of 128 Pa on cell surface (erythrocytes) has been derived from finite element method simulations considering the full microfluidic geometry. Statistical significances have been calculated from linear mixed models and error bars represent standard error of the mean (*, p<0.05; **, p<0.01; ***, p<0.001).

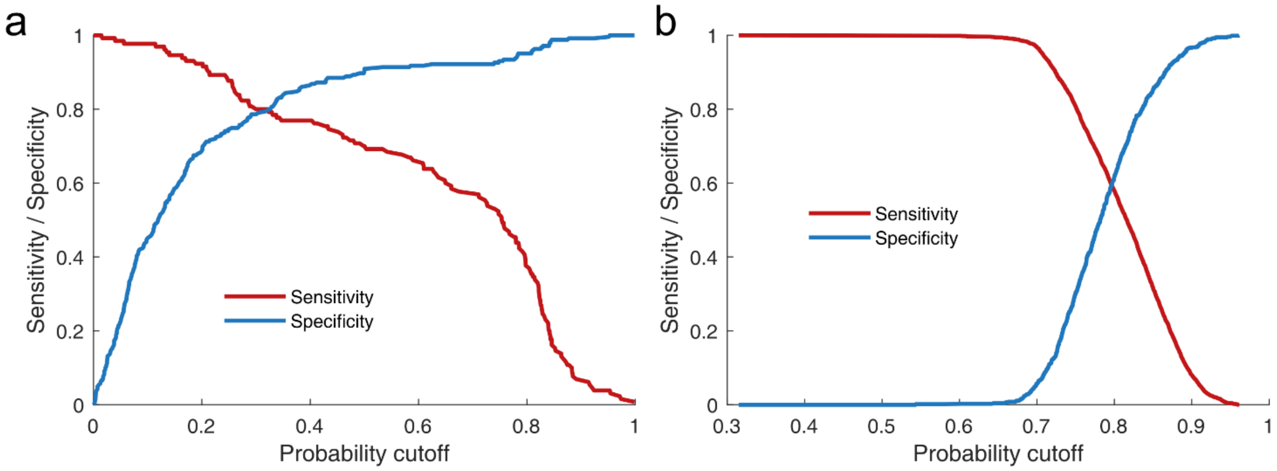

Supplementary Figure 7: Logistic model for primary blood cells. a) Sensitivity (red) and specificity (blue) for peripheral blood mononucleated cells (PBMCs) in a mixed population of granulocytes and PBMCs and b) sensitivity and specificity for CD4+ T-cells in a mixed lymphocyte population. Model selection using a multi-parameter approach was based on the Akeike information criterion (AIC) analyzing cell size, Young's modulus and viscosity for (a) and cell size, peak deformation at the inlet and inlet relaxation time for (b). For (a) a model incorporating cell size and viscosity yields the lowest AIC (area under the curve AUC=0.88 and probability cutoff 0.32), while the minimum in AIC for (b) is found for a two-parametric model of the peak deformation at the inlet and the inlet relaxation time (AUC=0.62 and probability cutoff 0.80) from the receiver operating characteristic.

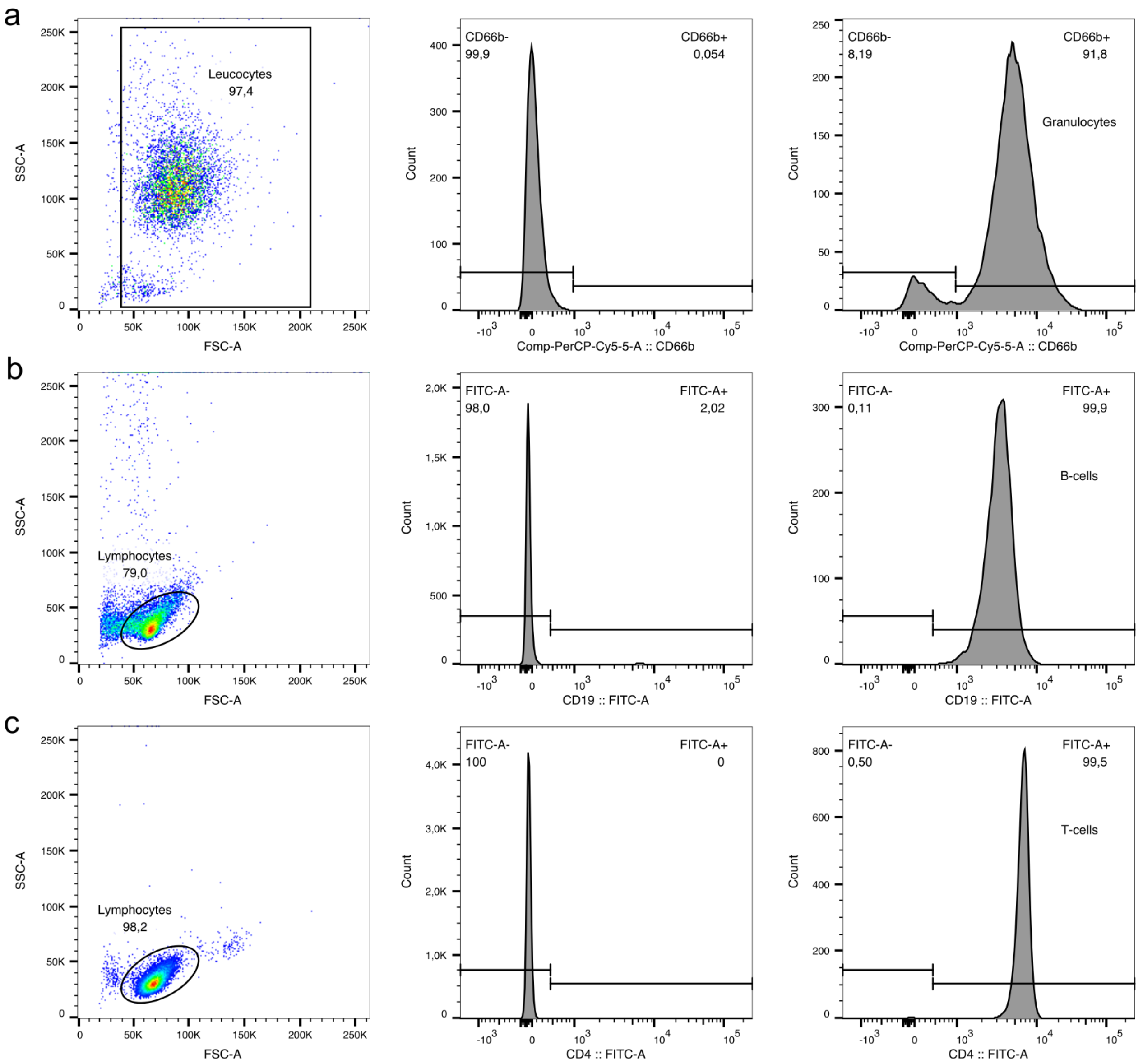

Supplementary Figure 8: FACS analysis of sample purity. Sideward vs. forward scatter plot for stained (left), negative control (middle) and positive control (right) for a) CD66b+ Granulocytes stained with PerCP-Cy5.5, b) CD19+ B-cells stained with FITC and c) CD4+ T-cells stained with FITC.

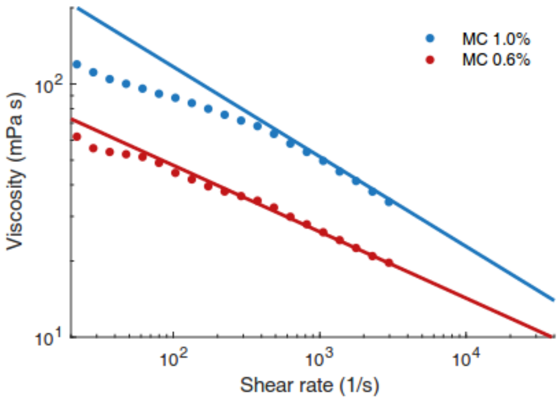

Supplementary Figure 9: Shear rate dependent viscosity of measurement buffer. Shear thinning behaviour quantified by rheometer (MCR 502, Anton Paar) using a cone-plate system (modules CP50-2/TG and P-PTD200/TG, Anton Paar). Measurement buffer consists of 0.6 % (w/v, red) or 1.0 % (w/v, blue) methylcellulose (MC 1.0 % and MC 0.6 %), respectively, dissolved in PBS-/- buffer. Blue and red lines indicate a power-law fit to the relevant shear-rate range.

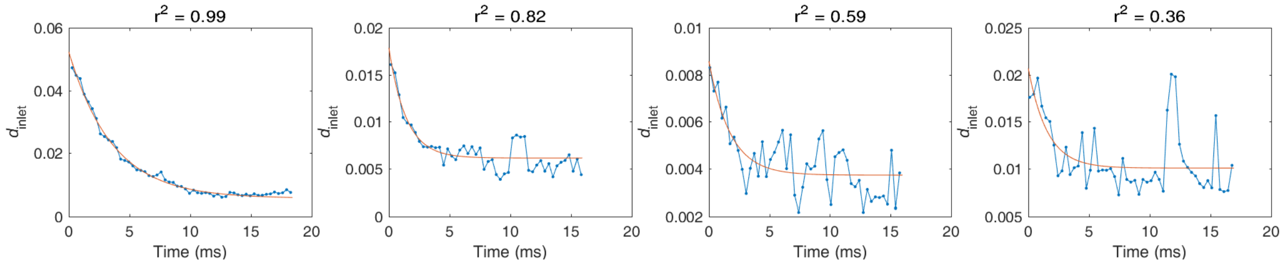

Supplementary Figure 10: Coefficient of correlation for exponential fit. Four examples showing the coefficient of correlation r^2 after fitting an exponential function (red solid line) to d_{inlet} versus time trace (blue) of HL60 cells in a 30 µm x 30 µm channel at a flow rate of 8 nl s^{-1}. For data analysis only fits with r^2=0.6 or higher have been considered.

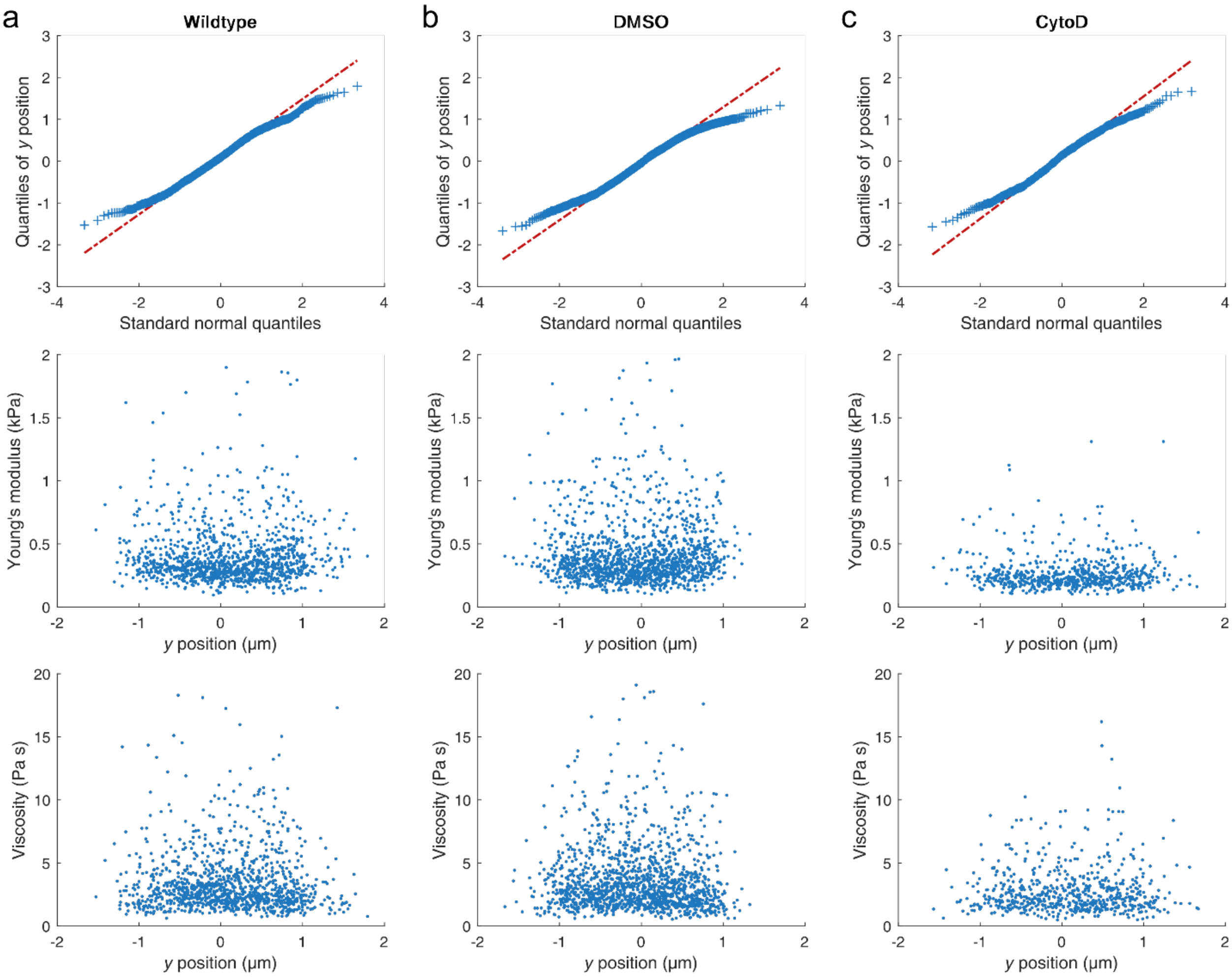

Supplementary Figure 11: Lateral position dependency of Young's modulus and viscosity. Q-Q plots and scatter plots of apparent Young's modulus as well as apparent viscosity as function of lateral channel position at outlet for a) wild type cells (*n* = 1,167), b) DMSO control (0.25 % (v/v), *n* = 1,436) and c) after treatment with 1 µM Cyto D (*n* = 654) from data shown in Fig. 3. Spearman rank correlation coefficient between apparent Young's modulus and lateral channel position has been determined to (a) ϱ = 0.029 (wildtype cells), to (b) ϱ = 0.051 (DMSO control) and to (c) ϱ = 0.018 (CytoD treatment). Between the apparent viscosity and the lateral channel position the Spearman rank correlation coefficient has been determined to (a) ϱ = -0.054 (wild type cells), (b) ϱ = -0.089 (DMSO control) and (c) ϱ = -0.029 (CytoD treatment). Dashed-dotted red line indicates a normal distribution for reference in (a). Measurements have been carried out using HL60 cells in a 30 µm x 30 µm channel at a flow rate of 8 nl s^{-1}. The mean shear rate of 5,100 s^{-1} and the mean stress of 142 Pa on cell surface has been derived from finite element method simulations considering the full microfluidic geometry.

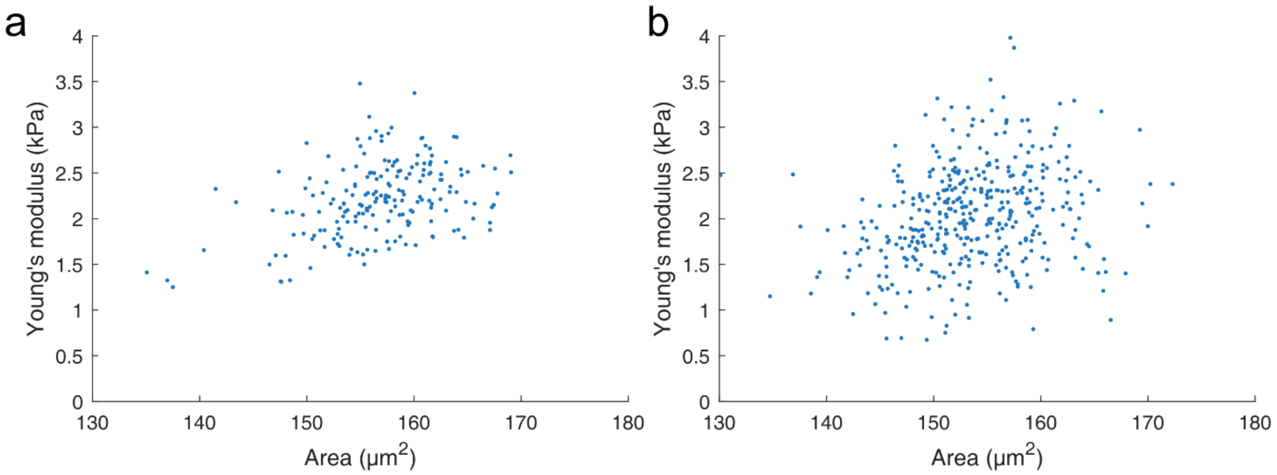

Supplementary Figure 12: Rheology on poly-acrylamide microgel elastic beads. a) Scatter plot of apparent Young's modulus as function of bead size for 199 beads measured in a 20 µm x 20 µm channel at a flow rate of 16 nl s^{-1}. The mean shear rate of 47,800 s^{-1} and a mean shear stress of 639 Pa on bead surface has been derived from finite element method simulations considering the full microfluidic geometry. Median apparent Young's modulus has been calculated to 2.20±0.03 kPa and median characteristic times to τ_{inlet} = 0.36±0.01 ms and τ_{channel} = 0.56±0.05 ms. (b) Scatter plot of apparent Young's modulus as function of bead size for 412 beads measured in a 30 µm x 30 µm channel at a flow rate of 128 nl s^{-1}. The mean shear rate of 76,000 s^{-1} and a mean shear stress of 910 Pa on bead surface has been derived from finite element simulations considering the full microfluidic geometry. Median apparent Young's modulus has been calculated to 2.00±0.04 kPa and median characteristic times are τ_{inlet} = 0.12±0.01 ms and τ_{channel} = 0.48±0.21 ms. Specified uncertainties represent the standard error of the mean of the sample.

Parameter	AIC	Cutoff	AUC
(1) A	318.77	0.30	0.87
(2) E	466.54	0.32	0.62
(3) η	452.98	0.32	0.71
(4) $A + E$	314.38	0.30	0.88
(5) $A + \eta$	310.98	0.32	0.88
(6) $E + \eta$	447.37	0.30	0.73
(7) $A + E + \eta$	310.26	0.30	0.88

Supplementary Table 1: Parameter sets for logistic regression model for granulocytes and peripheral blood mononuclear cells. Logistic regression models have been tested for all combinations of cell size A, the apparent Young's modulus E and the apparent viscosity η.

Parameter	AIC	Cutoff	AUC
(1) A	3737.27	0.80	0.58
(2) $\hat{d}_{\text{inlet}}$	3650.66	0.80	0.62
(3) τ_{inlet}	3745.98	0.81	0.49
(4) $A + \hat{d}_{\text{inlet}}$	3649.43	0.80	0.62
(5) $A + \tau_{\text{inlet}}$	3736.54	0.80	0.57
(6) $\hat{d}_{\text{inlet}} + \tau_{\text{inlet}}$	3644.10	0.80	0.62
(7) $A + \hat{d}_{\text{inlet}} + \tau_{\text{inlet}}$	3643.52	0.80	0.62

Supplementary Table 2: Parameter sets for logistic regression model for B-cells and CD4+ T-cells. Logistic regression models have been tested for all combinations of cell size A, the peak inlet deformation $\widehat{d}_{\text{inlet}}$ and the inlet relaxation time τ_{inlet}.

Channel cross-section (μm^2)	Flow rate ($nl\ s^{-1}$)	Cell dia-meter (μm)	Shear rate (s^{-1})	Hydrodynamic shear stress (Pa)	Dynamic viscosity (mPa s)
20 * 20	4	6.7	9904	221	25.0
20 * 20	8	6.7	18854	335	19.9
20 * 20	16	6.7	40857	551	15.1
20 * 20	4	10.0	9744	216	26.0
20 * 20	8	10.0	21510	363	19.3
20 * 20	16	10.0	39201	531	15.8
20 * 20	4	13.3	11538	234	27.8
20 * 20	8	13.3	23680	373	21.3
20 * 20	16	13.3	44667	617	17.9
30 * 30	4	10.0	2629	94	40.0
30 * 30	8	10.0	5259	147	31.3
30 * 30	16	10.0	10518	230	24.4
30 * 30	4	15.0	2621	93	42.0
30 * 30	8	15.0	5105	142	33.1
30 * 30	16	15.0	10243	223	25.9
30 * 30	4	20.0	2898	95	49.4
30 * 30	8	20.0	5864	149	38.2
30 * 30	16	20.0	11728	234	29.8

Supplementary Table 3: Finite element method simulations of hydrodynamic flow profile around a cell. Calculations of shear rate, mean hydrodynamic shear stress and viscosity have been carried out for channel cross-sections of 20 μm x 20 μm and 30 μm x 30 μm, varying flow rates and different cell diameters under steady-state conditions.
