# Supplementary material for: High-throughput single-cell rheology in complex samples by dynamic real-time deformability cytometry
Source: Nat Commun. 2019 Jan 24;10:415. doi: 10.1038/s41467-019-08370-3 (PMC6346011; doi:10.1038/s41467-019-08370-3)
Supplement: Supplementary file 3 — Reporting Summary Checklist [file 41467_2019_8370_MOESM3_ESM.pdf]

nature research

Corresponding author(s): Oliver Otto

Reporting Summary

Nature Research wishes to improve the reproducibility of the work that we publish. This form provides structure for consistency and transparency in reporting. For further information on Nature Research policies, see Authors & Referees and the Editorial Policy Checklist.

Statistical parameters

When statistical analyses are reported, confirm that the following items are present in the relevant location (e.g. figure legend, table legend, main text, or Methods section).

n/a	Confirmed	
☐	☒	The exact sample size (n) for each experimental group/condition, given as a discrete number and unit of measurement
☐	☒	An indication of whether measurements were taken from distinct samples or whether the same sample was measured repeatedly
☐	☒	The statistical test(s) used AND whether they are one- or two-sided<br>*Only common tests should be described solely by name; describe more complex techniques in the Methods section.*
☒	☐	A description of all covariates tested
☐	☒	A description of any assumptions or corrections, such as tests of normality and adjustment for multiple comparisons
☐	☒	A full description of the statistics including central tendency (e.g. means) or other basic estimates (e.g. regression coefficient) AND variation (e.g. standard deviation) or associated estimates of uncertainty (e.g. confidence intervals)
☐	☒	For null hypothesis testing, the test statistic (e.g. F, t, r) with confidence intervals, effect sizes, degrees of freedom and P value noted<br>*Give P values as exact values whenever suitable.*
☒	☐	For Bayesian analysis, information on the choice of priors and Markov chain Monte Carlo settings
☒	☐	For hierarchical and complex designs, identification of the appropriate level for tests and full reporting of outcomes
☒	☐	Estimates of effect sizes (e.g. Cohen's d, Pearson's r), indicating how they were calculated
☐	☒	Clearly defined error bars<br>*State explicitly what error bars represent (e.g. SD, SE, CI)*

Our web collection on statistics for biologists may be useful.

Software and code

Policy information about availability of computer code

Data collection	Data acquisition was carried out using a custom Labview script as outlined in the Methods section. The script is available on request.
Data analysis	Data analysis has been done using COMSOL 5.3a (finite element method simulations) and Matlab R2017a (analysis of cell shape modes and analytical model) as outlined in the Methods section. The COMSOL mesh files and the Matlab script are available on request.

For manuscripts utilizing custom algorithms or software that are central to the research but not yet described in published literature, software must be made available to editors/reviewers upon request. We strongly encourage code deposition in a community repository (e.g. GitHub). See the Nature Research guidelines for submitting code & software for further information.

Data

Policy information about availability of data

All manuscripts must include a data availability statement. This statement should provide the following information, where applicable:

- Accession codes, unique identifiers, or web links for publicly available datasets
- A list of figures that have associated raw data
- A description of any restrictions on data availability

The datasets generated in this study are available from the corresponding author upon reasonable request. The file format of the raw data is TDMS which can be read and analysed with Matlab or ShapeOut.

Field-specific reporting

Please select the best fit for your research. If you are not sure, read the appropriate sections before making your selection.

☒ Life sciences ☐ Behavioural & social sciences ☐ Ecological, evolutionary & environmental sciences

For a reference copy of the document with all sections, see nature.com/authors/policies/ReportingSummary-flat.pdf

Life sciences study design

All studies must disclose on these points even when the disclosure is negative.

Sample size	Sample size for experiments on cell lines was determined by monitoring convergence of median deformation value. In general, sample sizes of approximately n=1,000 have been proven to be sufficient.<br>For primary cells smaller sample sizes have been chosen to reduce the measurement time. Extended data acquisition duration due to low cell concentrations potentially induces artifacts by alterations of cells in suspension.
Data exclusions	For data analysis a convex contour filter has been applied.<br>Quantification of cell deformation relies on cellular perimeter and area and requires fitting a contour to each cell. Quality of the fit is determined by comparing the area inside the contour to the actual cell area. Data has been excluded if both parameters deviate more than 20% (erythrocytes), more than 10% (granulocytes) and more than 5% (PBMCS and cell line).<br>For quantifying cellular viscosity an exponential function has been fit to our data. Quality of the fit is determined by the coefficient of correlation where traces with r^2<0.6 have been excluded.<br>In Histograms of Figure 3b) and c) data points outside a confidence interval of 3*sigma are considered outliers.
Replication	Measurements have been carried out for biological replicates and / or on separate days.
Randomization	Not relevant as experiments have been carried out on primary cells and cell line.
Blinding	Not relevant as experiments have been carried out on primary cells and cell line.

Reporting for specific materials, systems and methods

Materials & experimental systems

n/a	Involved in the study	
☒	☐	Unique biological materials
☐	☒	Antibodies
☐	☒	Eukaryotic cell lines
☒	☐	Palaeontology
☒	☐	Animals and other organisms
☒	☐	Human research participants

Methods

n/a	Involved in the study	
☒	☐	ChIP-seq
☐	☒	Flow cytometry
☒	☐	MRI-based neuroimaging

Antibodies

Antibodies used	Antibodies have been used to quantify purity of B- and CD4+ T-cells after isolation. The following antibodies from Biolegend have been used:<br>Granulocytes: PerCP/Cy5.5 CD66b, G10F5, LOT: B159853, CAT: 305108<br>CD4+ T-cells: FITC CD4, OKT4, LOT: B164793, CAT: 317408<br>B-cells: FITC CD19, HIB19, LOT: B150301, CAT: 302206
Validation	Antibodies have been validated by the manufacturer in flow cytometry experiments

Eukaryotic cell lines

Policy information about cell lines

Cell line source(s)	HL60 cell lines as courtesy of Don and Ada Olins. They established the cell line.
Authentication	Authentication by Don and Ada Olins

Mycoplasma contamination

Cell lines were tested negative for mycoplasma contamination as stated in the Methods section

Commonly misidentified lines
(See ICLAC register)

Name any commonly misidentified cell lines used in the study and provide a rationale for their use.

Flow Cytometry

Plots

Confirm that:

- [x] The axis labels state the marker and fluorochrome used (e.g. CD4-FITC).
- [x] The axis scales are clearly visible. Include numbers along axes only for bottom left plot of group (a 'group' is an analysis of identical markers).
- [x] All plots are contour plots with outliers or pseudocolor plots.
- [x] A numerical value for number of cells or percentage (with statistics) is provided.

Methodology

Sample preparation

after purification, antibody stained for 30 min at room temperature in dark environment, 2 washing steps

Instrument

LSRII

Software

BD FACSDiva 6.2

Cell population abundance

Granulocytes 91.8 %, B-cells 99.9 % and T-cells 99.5 %

Gating strategy

FSC/SSC for complete cell population gated on lymphocytes / granulocytes, histogram over cell count for CD4 / CD19 / CD66b positive cells, negative control for unstained cells

- [x] Tick this box to confirm that a figure exemplifying the gating strategy is provided in the Supplementary Information.
